# Association Between Hepatitis C and Hepatocellular Carcinoma

**DOI:** 10.4103/0974-777X.52979

**Published:** 2009

**Authors:** Luis Jesuino de Oliveria Andrade, Argemiro D'Oliveira, Rosangela Carvalho Melo, Emmanuel Conrado De Souza, Carolina Alves Costa Silva, Raymundo Paraná

**Affiliations:** *Department of Medicine of Federal University of Bahia, Brazil*; 1 *Department of Faculty of Medicine of State University of Santa Cruz, Bahia, Brazil*

**Keywords:** Hepatocellular carcinoma, Hepatitis C virus, Hepatitis C

## Abstract

Hepatocellular carcinoma (HCC) is the fifth most common cancer, the third most common cause for cancer death in the world, a major cause of death in patients with chronic hepatitis C virus infection, and responsible for approximately one million deaths each year. Overwhelming lines of epidemiological evidence have indicated that persistent infection with hepatitis C virus (HCV) is a major risk for the development of HCC. The incidence of HCC is expected to increase in the next two decades, largely due to hepatitis C infection and secondary cirrhosis, and detection of HCC at an early stage is critical for a favorable clinical outcome. Potential preventive strategies in the development of HCC are being recognized. The natural history of HCC is highly variable and the clinical management choices for HCC can be complex, hence patient assessment and treatment planning have to take the severity of the nonmalignant liver disease into account. This review summarizes the inter-relationship between HCV and liver carcinogenesis.

## INTRODUCTION

Hepatocellular carcinoma (HCC) is the fifth most common cancer, the third most common cause for cancer death in the world,[1] the major cause of death in patients with chronic hepatitis C virus infection,[2] and responsible for approximately one million deaths each year.[3] Overwhelming lines of epidemiological evidence have indicated that persistent infection with hepatitis C virus is a major risk for the development of HCC.[4] The incidence of HCC is expected to increase in the next two decades, largely due to hepatitis C infection and secondary cirrhosis,[5] and detection of HCC at an early stage is critical for a favorable clinical outcome. Potential preventive strategies in the development of HCC are being recognized. Novel molecular markers identified may aid in the diagnosis of early HCC in patients with chronic HCV.[6]

Hepatitis C virus is the major causative agent of HCC, mainly through indirect pathways: Chronic inflammation, cell deaths, and proliferation. Clinical research using transgenic mouse models, in which the core protein of HCV has an oncogenic potential, indicate that HCV is directly involved in hepatocarcinogenesis.[7] Recent data has shown that HCV is capable of inducing this active production of free radicals per se, and it is not just through inflammation, a feature peculiar to this virus and the specific activity of its core protein.[8]

A further insight into the pathogenesis of HCC, as to how pathogenesis of an immune-mediated liver cell injury triggers the development of HCC in the absence of viral transactivation, and the molecular pathogenesis that overproduces the virus, largely envelopes the polypeptide and accumulates toxic quantities of the surface antigen within the hepatocyte, develop a severe, prolonged hepatocellular injury that initiates a programmed response within the liver, characterized by inflammation, regenerative hyperplasia, transcriptional deregulation, and aneuploidy progresses to neoplasia.[9]

The natural history of HCC is highly variable, the clinical management choices for HCC can be complex and thus patient assessment and treatment planning have to take the severity of the nonmalignant liver disease into account. The presence of cirrhosis usually places constraints on resection surgery, ablative therapies, and chemotherapy. Therefore, aggressive surgery or liver transplantation may be successful in treating small or slow-growing tumors if they are diagnosed early. Although chemotherapy and radiation treatments are not usually effective, they may be used to shrink large tumors so that surgery has a greater chance of success.

## HEPATITIS C

Hepatitis C virus infection was first suspected in the 1970s, when most blood transfusion infections were associated with either hepatitis A or hepatitis B virus. This new type of hepatitis transmitted by blood was then called “non-A, non-B” hepatitis. The genome of HCV was identified in 1989, and the name hepatitis C was subsequently applied to the human infection caused by this single-strand ribonucleic acid (RNA) virus of positive polarity.[10] Hepatitis C virus belongs to the Hepacivirus genus, *Flaviviridae* family, and has six major genotypes, and more than 70 subtypes.[11]

Hepatitis C virus has a major impact on public health, infects around 170 million people in the world, with an estimated global incidence of three to four million new infections per year.[12] Most patients infected with HCV are unaware of their exposure and remain asymptomatic during the initial stages of the infection; making early diagnosis during the acute phase (first six months after infection) is unlikely. While some of these infections will have a spontaneous resolution, the majority will progress to chronic HCV.[13] The infection by HCV may be unresolved in approximately 85% of the infected individuals, representing an important cause of liver cirrhosis and hepatocellular carcinoma.

The knowledge of the natural history of hepatitis C is still incomplete, because the acute infection is often asymptomatic in many individuals, as demonstrated in the epidemiological studies involving HCV infection and hemotherapy centers.[14] Moreover, this clinical form of HCV infection may present different geographical characteristics, which may be associated with ethnical / race and environmental factors such as HCV genotype and coinfection with other pathogenic agents.[15] The estimated mortality due to acute hepatitis C is very low (≤0.1%), contrasting with that verified for chronic HCV infection.

The transmission of HCV is mainly caused by infected blood or its products. However, other risks have been demonstrated for HCV infection, which are mainly represented by the intravenous use of illicit drugs, transplantation of HCV contaminated organs, and hemodialysis. Less frequent HCV infection has been documented as being due to occupational exposure to contaminated blood. Sporadic reports of HCV infection due to household exposure, vertical transmission, unsafe sex, and intranasal cocaine use, have also been published [16] However, the use of both third generation anti-HCV immunoassays and polymerase chain reaction to detect HCV-RNA in blood donors provoked an important fall in HCV infection, which has today an estimated incidence ratio of 1: 500,000-2,000,000 transfusions.[17]

HCV is the most common cause of chronic liver disease and cirrhosis in the world, and represents the main cause of liver transplantation in the United States of America, Australia, and Europe. The chronic infection is evidenced by the demonstration of HCV-RNA in the blood for at least six months after virus contamination.[14] Patients who exhibit clinical or laboratory signs of chronic liver disease must be diagnosed for chronic HCV infection through the demonstration of both anti-HCV antibodies and HCV-RNA.[18]

Besides hepatocytes, HCV infects different cells, including leukocytes and epithelial cells of different organs. However, it does not cause cytotoxicity, suggesting that both the hepatic injury and the extra-hepatic clinical manifestations caused by HCV infection are probably mediated by the immune events of cryoglobulinemia, immune complex persistence, and autoimmune recognition.[19] Thus, the pathogenesis of HCV infection involves a complex virus / host interaction.

## RELATION BETWEEN HEPATITIS C AND HEPATOCELLULAR CARCINOMA

Hepatocellular carcinoma accounts for 85 to 90% of the cases of primary liver cancer. Chronic hepatitis and cirrhosis constitute the major preneoplastic conditions in the majority of HCC. The risk of developing HCC for a patient with HCV-related cirrhosis is approximately 2-6% per year.[20] HCC risk increases to 17-fold in HCV-infected patients compared to HCV-negative subjects.[21] In general, HCC develops only after two or more decades of HCV infection and the increased risk is restricted largely to patients with cirrhosis or advanced fibrosis.[22]

Multiple steps are required in the induction of all cancers; it would be mandatory for hepatocarcinogenesis that genetic mutations accumulate in the hepatocytes. In HCV infection, however, some of these steps might be skipped in the development of HCC, in presence of the core protein. The overall effects achieved by the expression of the core protein would be the induction of HCC, even in the absence of a complete set of genetic aberrations, required for carcinogenesis. By considering such a non-Vogelstein type process for the induction of HCC, a plausible explanation might be given for many unusual events happening in HCV carriers.[23]

It is well known that the incidence of HCC in patients with HCV correlates with the progression of liver fibrosis. The degree of liver fibrosis and the time of acquisition of the infection modify the risk of HCC occurrence and are different in all HCV patients.[24] HCC without cirrhosis in HCV-infected patients, though rare, has been reported. The relation of the virus to the development of HCC is through chronic hepatitis and cirrhosis.

### Chronic hepatitis cirrhosis adenoma hepatocellular carcinoma

Hepatocyte necrosis and mitosis of chronic hepatitis favor nodular regeneration, which in appropriate circumstances, is followed by hepatocyte dysplasia and carcinoma.[25]

In many parts of the world HCC is among the leading causes of cancer-related mortality, and the third most common cause of cancer death in the world.[1,7] Japan, for example, unlike other Asian countries, also has a high proportion of HCC caused by HCV infection accounting for 80 to 90% of all cases,[26] while in the western world hepatocellular carcinoma is known to complicate cirrhosis secondary to hepatitis C in 2-6% per year.[20]

There is currently no evidence that HCV by itself is oncogenic; however, HCC may rarely develop in non-cirrhotic HCV-infected individuals, so a direct oncogenic effect cannot be excluded.[27] However, in the pathogenesis of HCC associated with HCV, it remains controversial whether the virus plays a direct or indirect role. Recent studies using transgenic mouse models, in which the core protein of HCV has an oncogenic potential, indicate that HCV is directly involved in hepatocarcinogenesis, albeit other factors such as continued cell death and regeneration associated with inflammation would also play a role.[7,28]

HCV causes HCC via an indirect pathway by causing chronic inflammation, cell death, proliferation, and cirrhosis.[7,29] HCV genomes can be detected in the tumor and surrounding liver tissue.[30] As for an association with the genotype of HCV and HCC, the incidence of genotype 1b is markedly high among the patients where it is associated with a more rapid deterioration of the liver histology in chronic hepatitis, although some studies have argued against this.[31]

A prospective study performed to establish whether infection with specific HCV genotypes was associated with an increased risk of development of HCC in cirrhosis, shows that cirrhotic patients infected with HCV type 1b carry a significantly higher risk of developing HCC than patients infected by other HCV types.[32]

There are suggestions that the presence of hepatitis B virus (HBV) gene in patients with chronic HCV-associated liver injury appears to promote hepatocarcinogenesis.[33] Similar to most types of cancer, hepatocarcinogenesis is a multistep process involving different genetic alterations that ultimately lead to the malignant transformation of the hepatocyte.[34]

In most patients with HCV-related HCC, the tumors are more likely to be solitary, smaller sized, and encapsulated, whereas, HBV-related HCC are more commonly infiltrative and multinodular.[35] The imaging diagnosis using ultrasound, computed tomography (CT), magnetic resonance, and hepatic angiography with or without CT is extremely useful for the detection of small HCC. Imaging diagnosis clearly indicates localization of the HCC inside the liver. However, confirmed diagnosis of a lesion is only available by the histological study of a biopsied specimen.[36]

Tumor markers are often used for screening of HCC. hepatoma tissues can synthesize various tumor-related proteins, polypeptides, and isoenzymes, such as alpha-fetoprotein (AFP), hepatoma-specific gamma-glutamyl transpeptidase (HS-GGT), and so on, and then secrete them into the blood. The valuable early diagnostic and prognostic biomarkers can predict the development and metastases of HCC. Recent researches have confirmed that circulating hepatoma-specific AFP subfractions, transforming growth factors (TGF)-β1, HS-GGT, and free insulin-like growth factors (IGF)-β1 may be more specific markers than the total AFP level for early diagnosis of HCC. Circulating genetic markers such as AFP-mRNA, TGF-β1-mRNA, IGF-II-mRNA, and so on, from the peripheral blood mononuclear cells of HCC patients have been most extensively used in monitoring distal metastasis or postoperative recurrence of HCC.[37]

Almost all HCC occurs in the liver of patients with chronic hepatitis and liver cirrhosis, caused by HBV and HCV. Consequently, eradication of these hepatitis viruses with anti-viral agents and chemoprevention methods may decrease the risk of HCC.[38]

Studies that have reported that *Interferon* (IFN) therapy, even after curative treatment for HCV-related HCC, could prevent HCC recurrence and improve survival,[39,40] despite the mechanisms by which IFN suppresses HCC recurrence, including possible direct anti-tumor and anti-inflammatory effects, remain uncertain.

Treatment options for HCC: Hepatectomy remains the standard treatment for HCC, but sufficient hepatic functional reserve is necessary.[41] Liver transplantation is the treatment of choice for patients with early HCC and decompensated cirrhosis; recent data have suggested that a modest expansion of tumor size limits could still preserve an acceptable, long-term, recurrence-free survival.[42] Local ablative therapy with percutaneous ethanol injection and radiofrequency ablation are most commonly used to destroy unresectable hepatic tumors.[43] Transarterial chemoembolization has been used for the majority with advanced disease. There is some evidence for survival benefit, but only in very carefully selected patients.[44] The systemic therapy using chemotherapy, immunotherapy, hormonal therapy or somatostatin analog have been disappointing in patients with advanced HCC.[45] Since HCC is a hypervascular cancer, gene therapy may be used for anti-angiogenesis to reduce tumor growth, and is a promising approach to treat HCC.[46]

HCC is a disease that requires multidisciplinary management: Gastroenterology / hepatology, surgery, transplant surgery, interventional and conventional radiology, medical oncology, radiation oncology, and nuclear medicine. Moreover, early diagnosis, correct treatment and referral to specialized services require a degree of high alert and an integration of all health services.

## CONCLUSION

At present, early diagnosis of HCC is critical for its effective treatment. The prognosis of untreated HCC is extremely poor, while therapeutic interventions are generally ineffective in advanced stages.[47] This review summarizes the inter-relationship between HCV and liver carcinogenesis.
